# Microbiome: A Nexus of One Health Strategies to Address Emerging Risks

**DOI:** 10.1093/biosci/biag011

**Published:** 2026-02-25

**Authors:** Damjana Drobne, Jerneja Ambrožič Avguštin, Mojca Bavcon Kralj, Jožica Čehovin Zajc, Jerneja Čremožnik Zupančič, Anna Dragoš, Andrej Ficko, Cene Fišer, Tjasa Griessler Bulc, Ester Heath, Matej Hočevar, Katja Hrovat, Darja Istenič, Sultana Jovanovska, Matic Kavčič, Anja Klančnik, Živa Zidar, Veronika Kralj Iglič, Andrej A Lukšič, Ines Mandic Mulec, Sara Novak, Valentina Perc, Tjaša Pogačar, Sonja Smole Možina, Urška Šunta, Polonca Trebše, Janez Vogrinc, Matej Vošnjak, Vesna Zupanc, Špela Železnikar, Marina Pintar

**Affiliations:** Department of Biology, Biotechnical Faculty, University of Ljubljana, Ljubljana, Slovenia; Department of Biology, Biotechnical Faculty, University of Ljubljana, Ljubljana, Slovenia; Biomedical Research Group, Faculty of Health Sciences, University of Ljubljana, Ljubljana, Slovenia; Faculty of Social Sciences, University of Ljubljana, Ljubljana, Slovenia; Faculty of Health Sciences, University of Ljubljana, Ljubljana, Slovenia; Department of Biology, Biotechnical Faculty, University of Ljubljana, Ljubljana, Slovenia; Department of Microbiology, Biotechnical Faculty, University of Ljubljana, Ljubljana, Slovenia; Department of Forestry and Renewable Forest Resources, Biotechnical Faculty, University of Ljubljana, Ljubljana, Slovenia; Department of Biology, Biotechnical Faculty, University of Ljubljana, Ljubljana, Slovenia; Faculty of Health Sciences, University of Ljubljana, Ljubljana, Slovenia; Faculty of Civil and Geodetic Engineering, University of Ljubljana, Ljubljana, Slovenia; Department of Environmental Sciences, Jožef Stefan Institute, Ljubljana, Slovenia; Institute of Metals and Technology, Ljubljana, Slovenia; Department of Biology, Biotechnical Faculty, University of Ljubljana, Ljubljana, Slovenia; Faculty of Health Sciences, University of Ljubljana, Ljubljana, Slovenia; Faculty of Civil and Geodetic Engineering, University of Ljubljana, Ljubljana, Slovenia; Faculty of Social Sciences, University of Ljubljana, Ljubljana, Slovenia; Faculty of Health Sciences, University of Ljubljana, Ljubljana, Slovenia; Faculty of Social Sciences, University of Ljubljana, Ljubljana, Slovenia; Faculty of Health Sciences, University of Ljubljana, Ljubljana, Slovenia; Department of Food Science and Technology, Biotechnical Faculty, University of Ljubljana, Ljubljana, Slovenia; Department of Food Science and Technology, Biotechnical Faculty, University of Ljubljana, Ljubljana, Slovenia; Biomedical Research Group, Faculty of Health Sciences, University of Ljubljana, Ljubljana, Slovenia; Faculty of Social Sciences, University of Ljubljana, Ljubljana, Slovenia; Faculty of Health Sciences, University of Ljubljana, Ljubljana, Slovenia; Department of Microbiology, Biotechnical Faculty, University of Ljubljana, Ljubljana, Slovenia; Department of Biology, Biotechnical Faculty, University of Ljubljana, Ljubljana, Slovenia; Department of Biology, Biotechnical Faculty, University of Ljubljana, Ljubljana, Slovenia; Department of Agronomy, Biotechnical Faculty, University of Ljubljana, Ljubljana, Slovenia; Department of Food Science and Technology, Biotechnical Faculty, University of Ljubljana, Ljubljana, Slovenia; Biomedical Research Group, Faculty of Health Sciences, University of Ljubljana, Ljubljana, Slovenia; Biomedical Research Group, Faculty of Health Sciences, University of Ljubljana, Ljubljana, Slovenia; Faculty of Education, University of Ljubljana, Ljubljana, Slovenia; Faculty of Education, University of Ljubljana, Ljubljana, Slovenia; Department of Agronomy, Biotechnical Faculty, University of Ljubljana, Ljubljana, Slovenia; Department of Agronomy, Biotechnical Faculty, University of Ljubljana, Ljubljana, Slovenia; Department of Agronomy, Biotechnical Faculty, University of Ljubljana, Ljubljana, Slovenia

**Keywords:** microbiome, metrices, indicators, citizen science, exposome

## Abstract

This paper positions microbiomes as pivotal to advancing the One Health framework, emphasizing their role in linking human, animal, and environmental health. Despite widespread support, implementation faces hurdles due to insufficient metrics, standardized indicators, and limited cross-sector collaboration. An interdisciplinary team investigates how climate change, pollution, and microbial contamination influence microbiome dynamics. Microbiome indicators like stress, resilience, and stability are proposed as proxies for health to guide evidence-based interventions. Key domains for microbiome assessment include the human and animal gut, soil, groundwater, and built environments, with food safety and water quality data serving as vital connectors. The paper introduces the nanostructurome to uncover microbial communication pathways and enable precision microbiome modulation. Integration of microbiological databases, microbiome literacy, citizen science, and social science perspectives is advocated to foster coordinated action. Ultimately, the microbiome is proposed as a strategic nexus for conceptual alignment, health indicator development, and targeted interventions.

Although the One Health approach is included in global commitments, policy statements, and the goals of international organizations, it has been criticized for its anthropocentric focus, overemphasis on emerging zoonotic diseases, inadequate integration of environmental concepts, and insufficient consideration of social science and behavioral aspects of health and governance (Manlove et al. 2016, Essack 2022, Miao et al. 2022). Within this One Health approach, ecosystem health is a crucial component. It refers to the ability of an ecosystem to maintain its structure, functions, and processes over time, while also being resilient to disturbance. It provides a stable foundation for maintaining biodiversity, supports vital ecological processes and provides services such as clean air and water, fertile soil for agriculture, and climate regulation (Fu et al. 2021). Understanding the intricate interactions between aquatic, soil, and microbial processes is fundamental to effective assessment and management of ecosystem health (Tecon and Or 2017, Zimnicki et al. 2020).

The authors of this paper have identified two main challenges with the current conceptualization of One Health. First, the absence of comprehensive indicators for environmental and ecosystem health hinders the accurate evaluation of human, animal, and ecological health, as well as the effectiveness of related interventions. Second, effective collaboration between researchers and sectors concerned with human, animal, plant, and environmental well-being is hindered by limited cross-sector coherence and insufficient integration of social sciences in discourse formation. In this paper, we address these challenges with the aim of enhancing our ability to tackle health issues in a more integrated and efficient manner. We propose microbiome as a strategic nexus for conceptual alignment, development of health indicators, and targeted interventions.

## Emerging risks affect microbiomes

In 2022, the Scientific Committee on Health, Environmental and Emerging Risks (SCHEER) published Statement II on Emerging Issues, which highlights various potential risks to human health and/or the environment in the future (Bertollini et al. 2022). These include climate change, water pollution, nanotechnology, chemical risks in a circular economy, degraded environment, and disease transmission.

In the following sections, we address two major environmental risks identified by SCHEER (2022), namely pollution and climate change, and discuss indicators of microbiome status as proxies for microbiome health, aimed at supporting informed decision-making within the One Health framework (figure 1).

**Figure 1.**
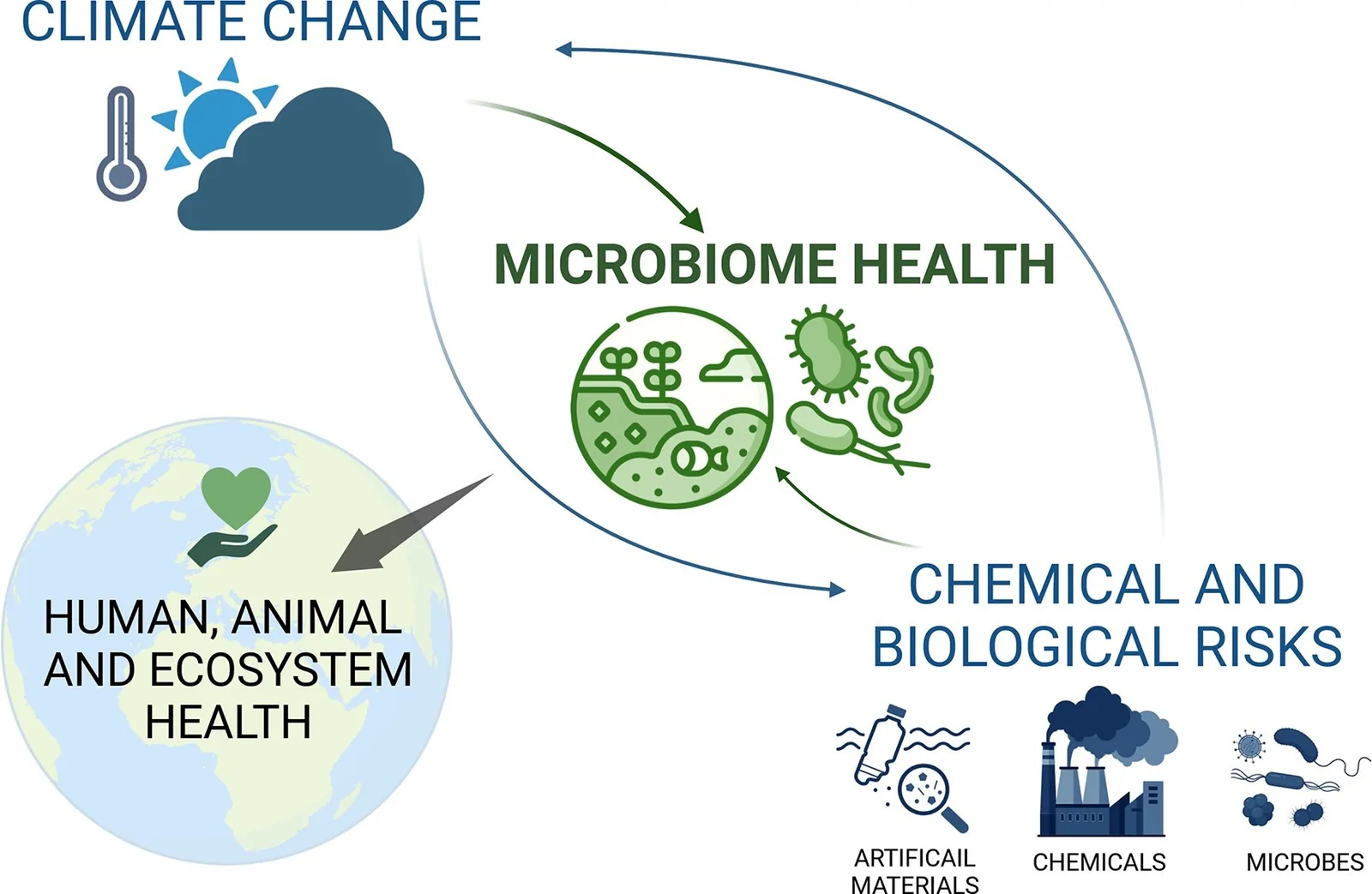
The interconnection between climate change, chemical and biological risks, human, animal, and ecosystem health (BioRender-generated image).

### Environmental pollutants change microbiome

#### Chemical pollution

Chemical pollution remains the world’s biggest environmental threat to human health, accounting for 15% of all deaths worldwide and 275 million disability-adjusted life years in 2017 (GAHP 2023). Exposure to environmental chemicals has been linked to various health disorders, including obesity, type 2 diabetes, cancer, and immune and reproductive system disorders. There is ample evidence that the gut microbiota is an important but underappreciated element that must be considered to fully assess the toxicity of environmental pollutants (Claus et al. 2016).

In addition, the use of bioindicator microorganisms has been recommended as a valuable strategy for analyzing the actual, direct, and timely effects of human activities on water, air, and soil. In particular, microbial indicators are used in the context of pathogenic microorganisms, as these can be transferred to the environment via feces (Choix et al. 2023).

#### Artificial material pollution (including nano- and microplastics)

Microplastics (MPs) are another ubiquitous pollutant that impairs environmental and food quality and subsequently affects the health of humans, animals, and ecosystems (Prata et al. 2021). The annual production of plastics continues to increase, from 460 million tons in 2019 to a projected 1231 million tons by 2060, with less than 10% of plastic waste being recycled. The rest either remains in landfills or accumulates in nature, where it is further degraded into micro- and nanoplastics (Li et al. 2022, Huang et al. 2024). According to the European Chemicals Agency definition, MPs include solid polymer particles up to 5 mm in size present in concentrations above 0.01%, whereas nanoplastics are plastic particles that are smaller than 100 nanometers in at least one dimension (Mitrano and Wohlleben 2020).

It is known that MPs can affect human and animal health by releasing potentially toxic chemicals, some of which are known as endocrine disruptors (bisphenols, phthalates, and per- and polyfluoroalkyl substances) (Trasande et al. 2024). In addition, plastic particles can serve as a vector for the transfer of pollutants (e.g., pesticides) into soil and water (De Souza Machado et al. 2019, Zhang et al. 2019). Another threat posed by plastic pollution is the formation of a unique microbial biofilm community that colonizes plastic debris, a plastisphere that can also act as a vector, allowing potentially harmful pathogens such as *Vibrio* spp., *Escherichia coli*, and bacteria carrying antibiotic resistance genes can spread across environments, even reach remote, pristine areas (Unveiling the Antarctic “plastisphere,” 2024, Caruso et al. 2024). The presence of potentially pathogenic bacteria and mobile genetic elements (i.e., antibiotic resistance genes) in the plastisphere is a major concern for the ecosystem and human health (figure 2) (Jaafarzadeh and Talepour 2023).

**Figure 2.**
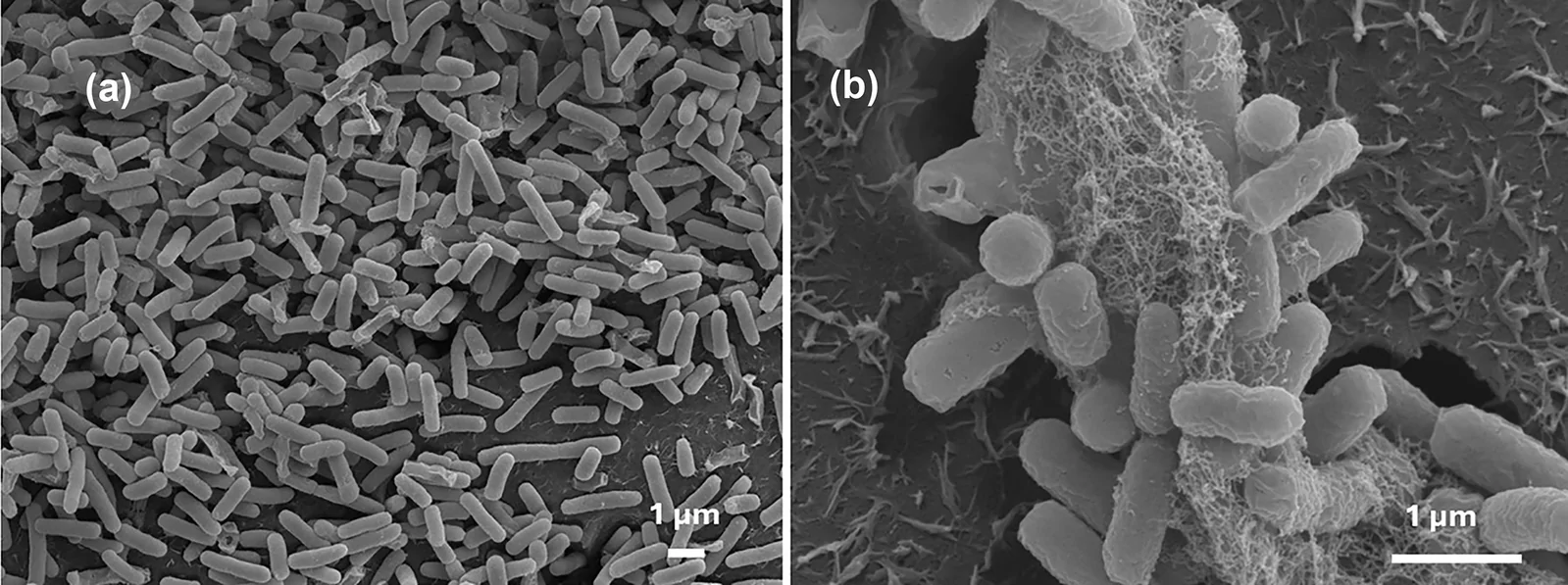
Power of scanning electron microscopy (SEM) to study bacteria on the surfaces. Images show *E. coli* on filter paper disk at lower (left) and higher (right) magnification (by Matej Hočever, IMT, Ljubljana).

#### Environmental microbial contamination

Microbial contamination also poses a significant threat to environmental health. Due to the risk of contamination by pathogenic bacteria, protozoa, and viruses, the sanitary integrity of aquatic environments, especially those used for drinking water supply or recreational activities, is of critical importance (Jung et al. 2014). In addition, antimicrobial resistance poses a significant danger to both human and animal health, antimicrobial resistance (AMR) is one of the greatest global threats to public health. It is estimated that bacterial AMR was directly responsible for 1.27 million deaths worldwide in 2019 and contributed to 4.95 million deaths (WHO 2023, CDC 2024).

### Climate change affects microbiomes directly and indirectly

#### Climate change as a part of exposome

Climate change is increasingly shaping the totality of exposures an individual or ecosystem faces over a lifetime (the exposome) and is additionally impacting both human and ecosystem health (Wild 2005, Abdelzaher et al. 2022, Do et al. 2024). Since there are interactions between climate change and chemical or artificial material pollutants or microbial contaminants, climate change should be interpreted as part of the exposome. According to Barouki (2024), toxicological evidence should be used to assess the risks associated with climate change (Barouki 2024).

#### Climate change transforms microbiomes

Climate change has a profound impact on microbial communities, which play an important role in ecosystems. Increased temperatures altered precipitation patterns, more frequent or intense extreme weather events such as heat waves, floods, and droughts, and increased use of reclaimed water are leading to shifts in microbial diversity, abundance, and activity that influence ecosystem processes (Lennon et al. 2023). For example, changes in soil temperature and moisture affect the diversity and activity of soil microorganisms (Jansson and Hofmockel 2020) and thus essential processes such as nutrient cycling, soil fertility, and plant growth (Jansson and Hofmockel 2020, Wanget al. 2024a). In addition, climate change also affects water temperature, pH, and oxygen levels, which in turn affects aquatic microbial communities and leads to a disruption of the balance of microbial processes such as decomposition and nutrient cycling. Microbes affect the global climate, and climate change affects microbes . Finally, microorganisms in the atmosphere, including those involved in cloud formation and precipitation, may also be affected by climate change. Changes in temperature and humidity can alter the distribution and activity of these microbes, potentially affecting weather patterns and precipitation cycles (Tiedje et al. 2022).

#### Climate change causes secondary risks

The implementation of several strategies is essential for effective adaptation to climate change. These include sustainable water management and resilient agricultural practices, which are paramount for sustainable use of natural resources and maintaining food security (Cotera et al. 2024). These strategies may pose secondary risks to human, animal, and ecosystem health.

For example, sustainable water management includes optimizing water use, conserving water resources, maintaining the water cycle, and improving water quality (Srivastav et al. 2021). However, reclaimed water may contain pathogens such as bacteria, viruses, and parasites, which can contaminate crops and endanger the health of consumers. Residues of chemicals, such as pharmaceuticals and industrial pollutants, in reclaimed water can accumulate in soil, soil microfauna, and crops or be washed into surface and groundwater, which can affect food safety, drinking water quality, and human health (Maitra et al. 2022, Swan et al. 2024).

Resilient agricultural practices also include adopting climate-smart farming practices, crop diversification, and improving soil health (Srivastav et al. 2021). For example, agronomic measures such as tillage, crop diversification, fertilizer use, and water management increase agricultural productivity while changing the microenvironment of the field. These changes affect soil microbial communities by disrupting natural ecosystems and introducing new microbial strains, which affect soil health (Souza et al. 2015, Adelisardou et al. 2023).

## Microbiome: a nexus of One Health

Historically, the field of microbiome research has emerged from environmental microbiome research (microbial ecology) and provides an interdisciplinary platform for different fields, for example, agriculture, food science, biotechnology, and medicine. Microbiomes play a crucial role as a bridge that facilitates the interactions and interdependence between humans, animals, plants, and the environment (Berg et al. 2020, Zhou et al. 2024, Muhummed et al. 2025). Consequently, there is a need to better understand these interactions to effectively prevent disease and manage health (Trinh et al. 2018, Muhummed et al. 2025). As a result, a new cross-sectoral approach focusing on the microbiome-centered One Health perspective is proposed and defined by Tomasulo and colleagues (2024) as “Microbiome One Health.” The authors present “Microbiome One Health” as an “efficient, holistic approach to the use of products and services that aims to maintain human well-being within a healthy ecosystem” (Tomasulo et al. 2024). Nevertheless, human health and the anthropocentric perspective predominate in the One Health microbiome perspective, and much less attention is paid to the environmental microbiome, such as the soil microbiome or the plant microbiome (figure 3).

**Figure 3.**
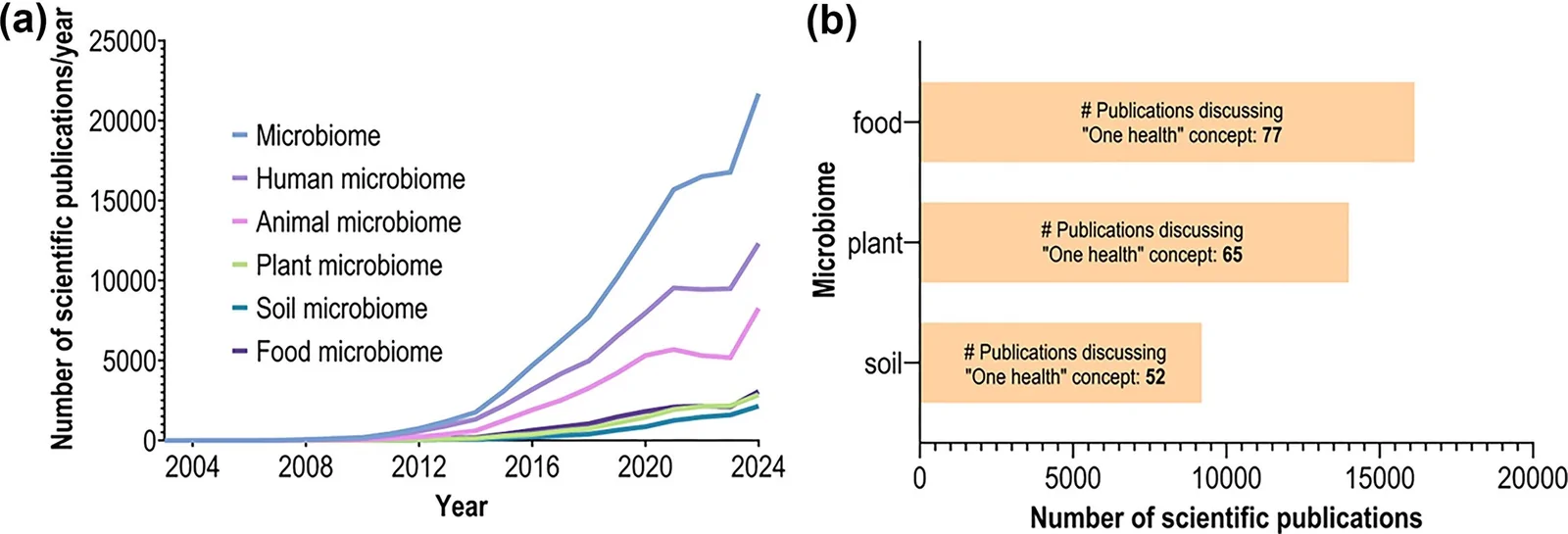
Production of microbiome-related scientific publications. (a) Annual production of scientific publications on microbiomes. (b) Total microbiome scientific publications; the number inside each horizontal column represents the number of studies also discussing “One Health.”

Figure 3 shows the underrepresentation of studies on the microbiome of plants, soils, and food. The data were retrieved from the Scopus database (https://www.scopus.com/) in January 2025 using the following terms in the search field “Article title, Abstract, Keywords”: microbiome; human microbiome; animal microbiome; food microbiome; plant microbiome; soil microbiome. The language was limited to English, and the publication years were from 2002 to 2024. The literature search for the three microbiomes was supplemented by an additional search term “One health” in the search field “Article title, Abstract, Keywords.” The number of publications that discuss “One Health” in connection with the respective microbiome is dominated by studies that focus on the human microbiome. The least attention is paid to the soil microbiome (figure 3).

### Soil microbiome health: the key component of ecosystem health

The Intergovernmental Technical Panel on Soils defines soil health as “the ability of the soil to sustain the productivity, diversity, and environmental services of terrestrial ecosystems” (FAO 2020). Healthy soil supports a vast array of organisms that contribute to the breakdown of organic matter and nutrient cycling. Moreover, healthy soil plays a critical role in water filtration, reducing runoff and erosion, and enhancing water retention (Marinari et al. 2022, Meersmans et al. 2024).

A healthy soil microbiome is a key prerequisite for soil health. It is characterized by soil fertility that promotes plant and animal growth, high microbial diversity, stable soil structure, resilience to disturbances, absence of contaminants, and active biogeochemical cycles that support soil carbon transformation and sequestration (Zimnicki et al. 2020, Banerjee and van der Heijden 2022). Microbes directly affect nutrient cycling, greenhouse gas fluxes, water purification, and organic matter accumulation, all of which can also influence human health and well-being. In addition, some soils act as a reservoir of antibiotic resistance genes (McEwen and Collignon 2018) but also of beneficial and pathogenic bacteria. Soil microbiomes have been also linked to soil suppressiveness, which can limit the spread of plant pathogens (Schlatter et al. 2017). Moreover, diversity of soil microbiomes has an inverse relationship with survival and spread of invading human pathogens *E. coli* O157:H7 (Van Elsas et al. 2012). However, assessing soil microbiome health is challenging due to the lack of universal standards and the variability in soil types, geographic locations, and human activities (Stewart et al. 2018, Choix et al. 2023). Consequently, soil health assessment remains largely dominated by chemical indicators (Lehmann et al. 2020, Willoughby et al. 2023). However, this dominance is beginning to shift, driven by growing recognition across scientific disciplines that soil health, and plant health are fundamentally linked to the conditions of the soil microbiome.

Calls are intensifying to incorporate soil health as a foundational element within One Health policy initiatives (Ohno and Hettiarachchi 2018, Andrivon et al. 2022, Banerjee and van der Heijden 2022, Muramoto et al. 2022, Singh et al. 2023, Montgomery et al. 2024, Morris et al. 2024). As a result, a Soil Security concept was developed that works under the human–animal–environment interface (Swan et al. 2024). There are five dimensions that frame the Soil Security concept referring to the biophysical aspects of soil, such as the capacity and condition, and the socioeconomic factors such as capital, connectivity, and codification. Swan and colleagues (2024) identified that, despite the interconnectedness of the Soil Security and One Health research fields and their focus on human–animal–environment interactions, these fields operate independently in separate domains, indicating a degree of compartmentalization or “siloing.” In practice, the two concepts are treated as separate entities with limited integration. Swan and colleagues (2024) highlight the Soil Health concept as a potential connector between Soil Security and One Health.

Soil health, defined through soil microbiome health, can indeed integrate the concepts of One Health and Soil Security.

### Food microbiome: pillars of One Health

By simple definition, the microbiome of food includes all the microorganisms present in food items, but according to the renewed definition, the microbiome includes both the microbiota (community of microorganisms) and the environmental conditions that support them and present their “theatre of activity.” The latter involves the whole spectrum of molecules produced by the microorganisms, including their structural elements (nucleic acids, proteins, lipids, polysaccharides), metabolites (signaling molecules, toxins, organic, and inorganic molecules), and molecules produced by coexisting hosts and structured by the surrounding environmental conditions (Berg et al. 2020). This food microbiome is directly dependent on plant, animal, or microbial raw materials, water and air quality, and other food-contact hygiene parameters in the food production and processing environment. The balance between beneficial and harmful microbes in these facilities directly influences food product microbiome, which has a crucial impact on food quality and safety (Bedale 2018).

Microbiomes in the food production and supply chains are indicators of hazards (e.g., pathogens, their virulence factors, and antimicrobial resistance). Consequently, microbiome data are included into food risk assessments as a key regulatory requirement, although the microbiome’s potential as a component of risk assessment is still limited due to knowledge gaps and the lack of a consensus for the definition of a “healthy microbiome” (Bronzwaer et al. 2019, FAO 2022). Still, this approach helps to predict and mitigate risks associated with foodborne pathogens and contaminants, enhancing the overall safety of the food supply (Bevilacqua et al. 2023, Imai et al. 2024).

In addition, food microbiomes play a key role in shaping our gut microbiome. For example, fermented foods with beneficial bacteria can enhance gut health by promoting a diverse but balanced gut microbiome. This is further linked to numerous other health benefits, including improved digestion, synthesis of vitamins, regulation of fat storage, enhanced immune function, and even protection of mental health or prevention against chronic conditions such as diabetes or obesity (Valdes et al. 2018).

There is another important aspect where microbiomes are related to diet. This includes live biotherapeutic products, dietary supplements, and foods containing live microorganisms (Therapeutics et al. 2024). The safety of food cultures is based on their history of safe use, absence of harmful metabolites, and compliance with regulatory guidelines such as those provided by European Food Safety Authority (EFSA; Rychen et al. 2018).

By understanding food safety as an integral part of the One Health principles, we help to ensure that food production systems are not only safe, but also sustainable and resilient. Only in this way, and with greater awareness among the various stakeholders, will it be possible to achieve food security for the world’s growing population, preserve natural resources, and improve health by ensuring food safety (Garcia et al. 2020).

### Microbiome of the built environment: ubiquitous and influential

The term microbiome of the built environment (MoBE) is frequently used to describe the intricate relationship between human health and the microbial communities present in residential and other built environments (Jayaprakash et al. 2017, Matthews et al. 2024). MoBEs are influenced by microbial sources such as humans and pets, as well as by microbes from external sources such as plants and soils. This dynamic interaction highlights the complexity and variability of microbial communities within built environments (Imai et al. 2024). Given that about 50% of the total human population lives in urban environments and that humans spend approximately 90% of their time indoors, the presence of microbial DNA in these spaces is significant. Studies have shown that 5%–40% of DNA sequences found in built environments are of microbial origin (Adams et al. 2016). In addition, humans shed approximately 30 million microbial cells each hour into their immediate environment, making humans the primary contributor to shaping the microbiome found within the built environment (Ursell et al. 2012).

Research in this field aims to better understand how the composition and function of the MoBE affect human health. For instance, certain beneficial microbes can enhance indoor air quality by breaking down pollutants, whereas others may promote colonization of pathogens. Conversely, an imbalance in the MoBE can contribute to the spread of infectious diseases and contribute to health risk.

In the context of the One Health perspective, MoBE emphasizes the importance of understanding how microbial communities in built environments interact with human and animal health, as well as the broader environment. For example, the One Health approach can help identify how changes in the built environment, such as improved ventilation or the use of antimicrobial surfaces, can influence the MoBE and subsequently affect human health. It also highlights the need for sustainable bioinformed design of buildings and urban areas that promote microbial diversity while minimizing exposure to harmful pathogens (Bruno et al. 2022).

By integrating microbial diversity into urban planning, cities can enhance ecosystem functions such as waste treatment, pollutant degradation, and nitrogen fixation, which are essential for maintaining a healthy environment (King 2014).

### Microbiome-based analyses: operationalizing One Health

One of the initial steps to enhance the operationalization of the One Health approach is to apply (One) health indicators that reflect the system states and trends (Zhang et al. 2022, Willoughby et al. 2023). Because microbiomes reflect anthropogenic disturbances, monitoring their changes enables early detection of environmental stress severity and potential health risks (Tiedje et al. 2022, Ribas et al. 2023, Wu et al. 2024).

#### Microbiological metrics

Basic microbiological metrics addressing health of humans, animals, and environment include microbial biomass, microbial community structure, presence or expression level of specific genes of interest, microbial enzyme activity, though these are not always reliable (Fierer et al. 2021). Despite the high potential of DNA-based analysis of microbial communities, this approach represents an under-utilized metric of environmental health. Techniques such as quantitative Polymerase Chain Reaction (PCR), amplicon sequencing (e.g., Internal Transcribed Spacer ITS for fungi or 16S rRNA gene for bacteria), and shotgun metagenomics offer transformative potential for measuring environmental health (Prosser and Sibley 2015). In addition, recent studies have demonstrated that information on soil bacterial community composition can predict key physico-chemical variables associated with soil quality (Hermans et al. 2020). Adopting microbes as bioindicators is a promising approach; however, it requires further validation of taxa or genes consistently associated with specific soil health traits. In the case of soil, these indicators should be integrated with well-characterized soil edaphic factors specific to different soil or ecosystem types (Hermans et al. 2017). The abovementioned microbiome-based approaches could be further advanced by metagenomics and metabolomics for in-depth characterizing environmental microbiomes.

In recent years, so-called synthetic microbial communities (SynComs) have emerged as a powerful tool for microbiome research, as they simplify natural microbiomes while preserving critical interactions, allowing deeper exploration of microbial dynamics under controlled conditions. SynComs play a crucial role in environmental remediation, encompassing enhanced bioremediation, MP degradation, and support in phytoremediation (Huang et al. 2024, Sharma et al. 2024).

In addition, scientists have made progress in identifying how soil microbiome structurome, such as the presence of specific microbial species and their changes, responds to various environmental interventions. However, a significant knowledge gap exists on how these structural changes translate into functional effects, both within environmental ecosystems and in their downstream impacts on human, animal, and plant health. Addressing this knowledge gap is critical for unlocking the full potential of microbiome monitoring and management strategies, enabling their integration into sustainable environmental practices and health policy (Singh et al. 2023).

Since microbiomes can influence food safety at multiple levels, EFSA has initiated exploratory projects for integration microbiome analyses in food safety assessments . Microbiome-based metrics for food safety and quality include microbial diversity assessments and pathogen detection using both culture-dependent and culture-independent approaches, particularly those utilizing next-generation sequencing technology. These metrics are crucial for ensuring food safety and quality in various food production environments (Sabater et al. 2021, Xu et al. 2025).

#### Microbiome-based indicators

Well-defined microbial community indicators, supported by standardized methods for their measurement and interpretation, are critical for generating reliable insights that inform evidence-based decision-making across health, agriculture, and environmental management (Galbraith Convertino 2021, Puig Ribas et al. 2023, Astudillo—García et al. 2019). Table 1 summarizes indicators of microbial community states, indicators definition, guides on how to measure the state of microbiome, and the results to be used for evidence-based decision-making, all supported by representative references.

**Table 1.** Microbial community indicators for evidence-based decision-making.

Microbial community indicators	Definition of indicator	How to measure (metrices)	How to use results to inform evidence-based decision-making	Representative reference
Stress	External/internal pressures that disrupt microbiome structure or function	Shifts in diversity, loss of key taxa	High stress may indicate vulnerability, dysbiosis, or environmental degradation	Rocca and colleagues (2019), Cheng and colleagues (2024)
Resilience	Ability to recover after disturbance	Longitudinal sampling, diversity recovery curves, functional restoration	High resilience suggests robust health or ecosystem stability, low resilience may signal chronic imbalance	Zhou and colleagues (2025)
Stability	Consistency of microbial composition/function over time	Time-series analysis, beta diversity, network stability metrics	Stable microbiomes are often linked to health. Instability may reflect disease or environmental stress	Kajihara and Hynson (2024)
Plasticity	Capacity to adapt or reorganize in response to change	Functional profiling (metagenomics, metabolomics), taxonomic shifts under stress	High plasticity may support adaptation but could also mask underlying instability	Brante and colleagues (2025)
Robustness	Resistance to change under pressure	Perturbation experiments, resistance to antibiotics or diet shifts	Robust microbiomes resist dysbiosis and maintain function under stress	Anthony and colleagues (2022)
Homeostasis	Balanced microbial state	Balanced taxa ratios, functional equilibrium, host biomarkers (e.g., inflammation)	Disruption of homeostasis may indicate disease onset or ecological imbalance	Lee and colleagues (2022)
Functional redundancy	Multiple taxa performing similar functions	Metagenomic pathway analysis, enzyme activity profiles	High redundancy buffers against functional loss, low redundancy increases fragility	Ramond and colleagues (2025)
Dysbiosis	Microbial imbalance associated with disease or degradation	Reduced diversity, overgrowth of pathogens, loss of beneficial microbes	Indicates compromised health or environmental stress; often linked to disease	Wei and colleagues (2021), Shen and colleagues (2025)
Recovery potential	Ability to return to a healthy state post-disturbance	Monitoring post-intervention changes, diversity and function restoration	High recovery potential supports resilience; poor recovery may require intervention	Jurburg and colleagues (2024)
Responsiveness	Speed and magnitude of microbiome reaction to stimuli	Time-course studies, functional shifts after diet, drugs, or environmental change	Rapid and appropriate response may indicate adaptability; exaggerated or delayed response may reflect dysfunction	Karwowska and colleagues (2024)

#### Monitoring microbial community indicators to support One Health

Microbial indicator monitoring plays a vital role in supporting the One Health framework by providing measurable insights into the health and stability of interconnected biological systems. These indicators, such as stress, resilience, homeostasis, dysbiosis, and recovery potential, enable scientists and policymakers to diagnose and monitor microbial health across ecosystems, from soil and water to the human and animal microbiomes. By tracking shifts in microbial diversity, function, and composition, they help predict the risk of disease emergence, ecological collapse, or antimicrobial resistance (table 1). In addition, microbial indicators are essential for tracing microbe transmission between humans, animals, and the environment, which is a core aspect of One Health. They enable early detection, prevention, and coordinated action across sectors (Couradeau et al. 2025).

There already exist monitoring programs that include microbiological analyses; however, these programs are often scattered, and their databases are not integrated (figure 4). In Europe, organizations like the EFSA and the European Centre for Disease Prevention and Control (ECDC) are actively involved in monitoring zoonotic diseases, antimicrobial resistance, and microbiological contaminants. The ECDC mission is to identify, assess, and communicate current and emerging threats to human health posed by infectious diseases. These monitoring programs are often criticized as they are focused on selected microorganisms only and not on a microbiome. Microbiome is monitored in the Earth Microbiome Project, which is systematically characterizing global microbial taxonomic and functional diversity of microbial life on this planet. They use DNA sequencing and mass spectrometry of crowd-sourced samples to understand patterns in microbial ecology across the biomes and habitats of our planet (Earthmicrobiome 2025).

**Figure 4.**
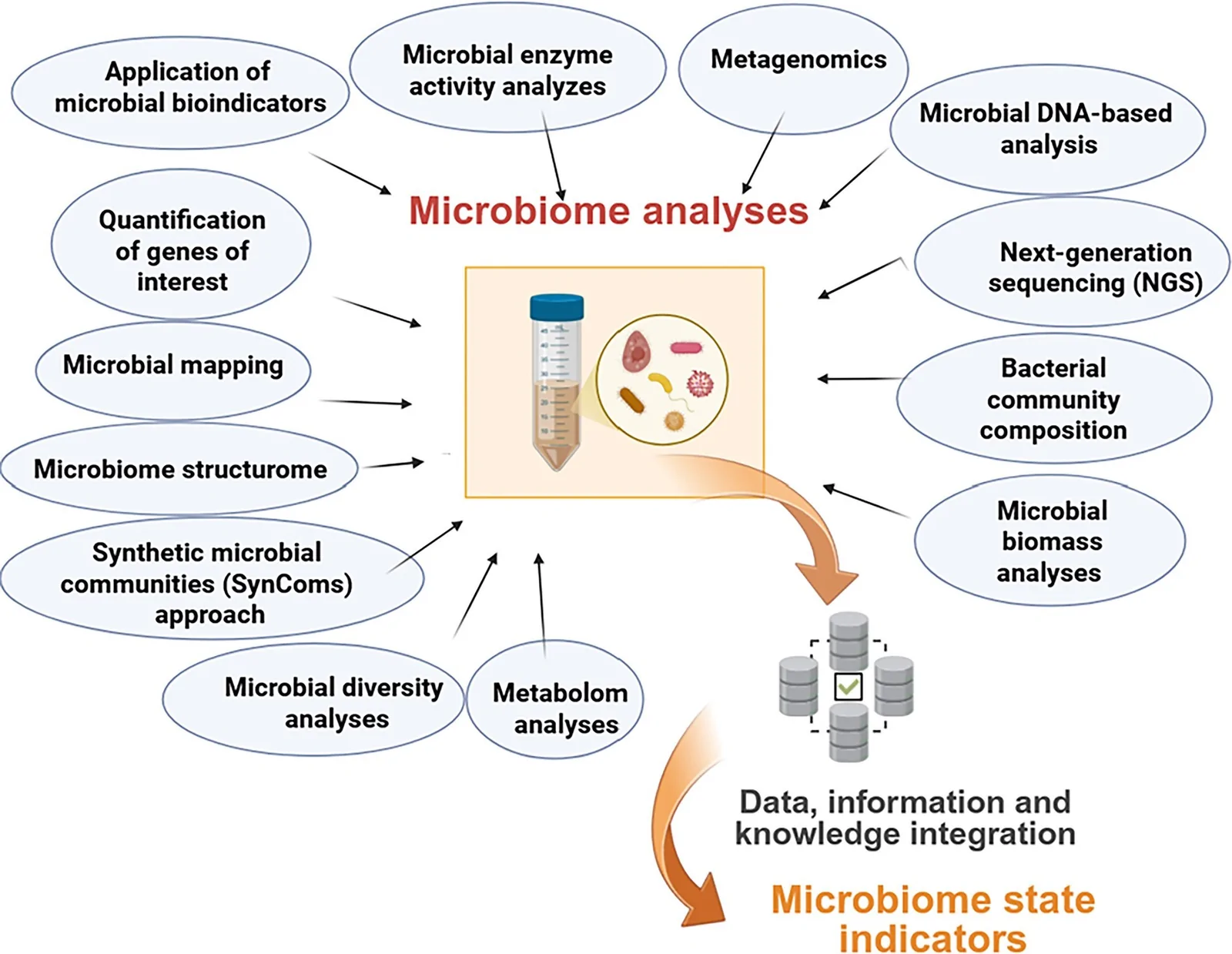
The integration of microbiological metrics into microbiome state indicators (BioRender-generated image).

Based on current evidence, microbial monitoring supports conservation and stewardship efforts while identifying microbial hotspots (Hampton-Marcell et al. 2023).

#### Microbiome-based interventions for global health

Insights from monitoring schemes inform the targeted interventions discussed in detail in the following chapters (figure 5).

**Figure 5.**
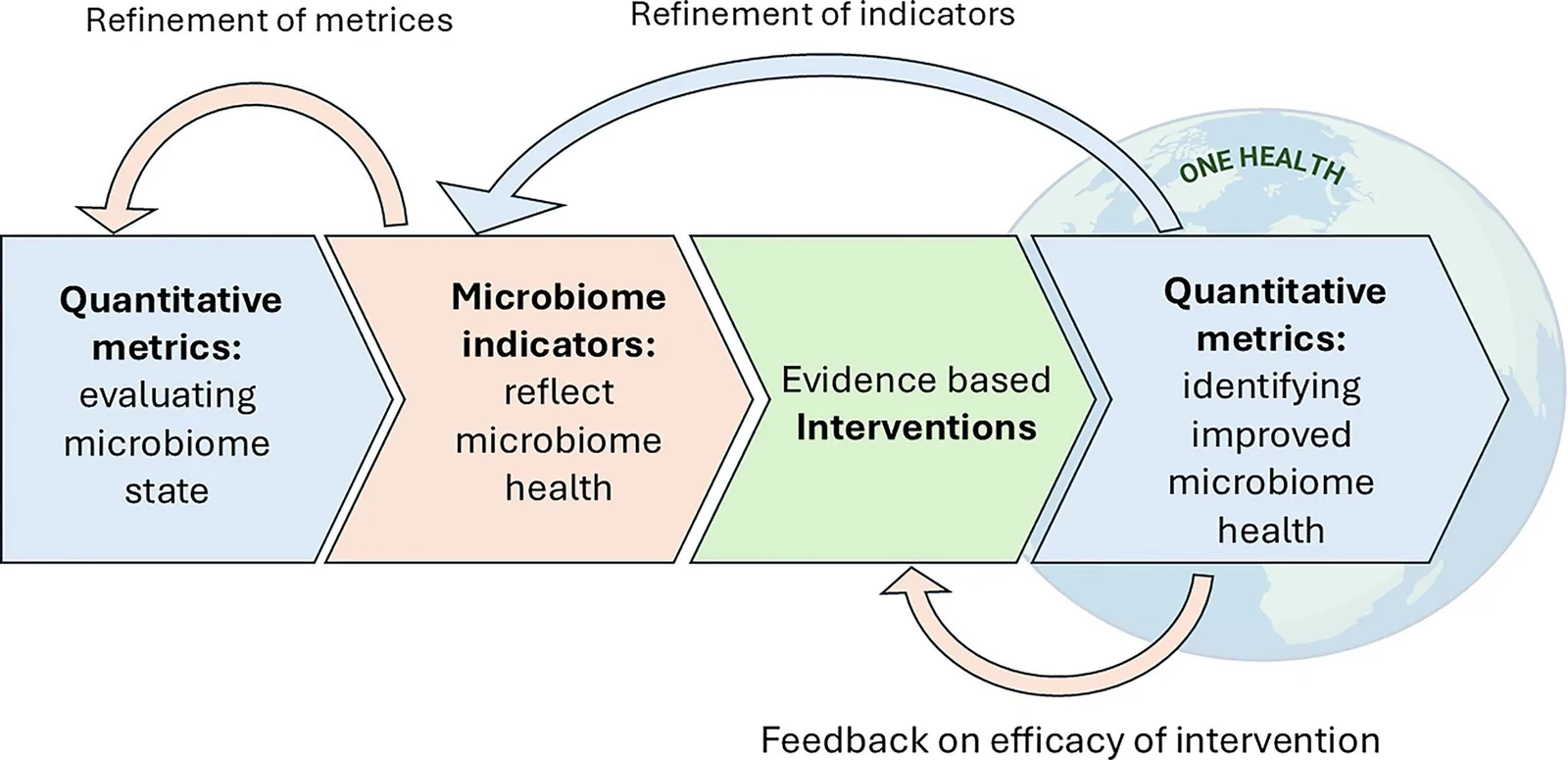
Quantitative metrics are integrated into indices that support informed decision-making for targeted interventions.

Restoring the microbiome after disturbance is a critical goal across human, animal, and environmental health domains. Within the One Health framework, several mitigation strategies have emerged that aim to reestablish microbial balance, function, and resilience.

##### Probiotic and prebiotic therapies for humans, animals, and plants

One of the most widely applied interventions to restore disturbed microbiomes is by probiotic and prebiotic supplementation (Saedi et al. 2025). Probiotics introduce beneficial microbial strains into the host or environment, whereas prebiotics provide substrates that selectively promote the growth of these microbes. This strategy has shown success in treating gastrointestinal disorders in humans, improving feed efficiency and immunity in livestock, as well as in stabilizing microbial communities in aquaculture systems.

For example, probiotic and prebiotic supplementation can be used to restore gut microbiota balance, reduce inflammation, and support immune function especially relevant in combating antimicrobial resistance and improving nutritional health (Saedi et al. 2025).

##### Phage therapy agents

After decades of abandonment, phage therapy is returning to the mainstream scientific acceptance (Petrovic Fabijan et al. 2023). Bacteriophages, often simply called phages, are viruses that infect and replicate within bacteria. In contrast to probiotics, phages are more specific, allowing to target only harmful bacteria, they are also self-amplifying and self-limiting, which reduces the risk of further microbiome disturbance (e.g., by overcolonization). For example, phage preparations targeting *Ralstonia solanacearum* have been successfully used against wilt disease, buffering the resident microbiota against changes caused by pathogen invasion (Wang et al. 2024b). Several studies demonstrated that phages could be used as biocontrol agents in livestock food production, targeting *Salmonella* spp., *E. coli, Campylobacter* spp., and *Clostridium* spp. (Gigante and Atterbury 2019, Gildea et al. 2022), and multiple clinical trials are ongoing for phage therapeutic in humans. Phage specificity reinforced personalized treatment, which requires a new regulatory framework—currently pioneered as so-called “magistral phage” in Belgium (Pirnay et al. 2018). Although phage therapy is still in the experimental stage, it represents a paradigm shift in microbiome-based interventions, paving the way for more personalized and precise modulation of individual microbiomes.

##### Fecal microbiota transplantation

In humans, fecal microbiota transplantation, particularly in clinical settings, and dietary modification are key strategies for microbiome restoration (Lee et al. 2024). As a lower-risk alternative, phageome transplantation has been employed, showing promising results in ulcerative colitis (Majzoub et al. 2024). This further confirms the strong potential of bacteriophages in microbiome-oriented interventions.

##### Microbiome-targeted therapies

In addition, microbiome-targeted therapies are gaining traction in precision medicine. These involve drugs or molecules designed to modulate specific microbial pathways or populations. Such therapies offer a more targeted approach to correcting dysbiosis and are being developed for conditions like inflammatory bowel disease, obesity, and even cancer (Pitashny et al. 2025).

##### Soil microbiome restoration and engineering

In environmental contexts, microbiome restoration often involves microbiome-assisted rehabilitation. This includes the use of microbial inoculants or engineered consortia to restore degraded ecosystems, such as coral reefs, polluted or degraded soils, or contaminated water bodies. These interventions aim to reintroduce functional microbial communities that can reestablish nutrient cycling, detoxification processes, and ecological resilience (Han et al. 2025).

Soil microbiome engineering is another emerging strategy in agriculture. By applying organic amendments, composts, or tailored microbial blends, practitioners can enhance soil health, promote plant growth, and increase carbon sequestration. This not only restores microbial balance but also contributes to climate mitigation efforts (Beattie et al. 2025).

Practices such as regenerative agriculture and composting enhance soil microbial diversity and also improve food security, climate resilience, and ecosystem health.

##### Microbiome-informed water treatment

Microorganisms play a vital role in the natural purification of water by breaking down organic pollutants, transforming harmful compounds, and outcompeting pathogenic species. Harnessing microbial communities to enhance water quality has emerged as a promising and increasingly adopted strategy in environmental management. Microbiome-informed water treatment is effective in both groundwater remediation and wastewater systems, where engineered or naturally occurring microbial consortia can be leveraged to reduce pathogen loads through competitive exclusion and predation, degrade contaminants such as nitrates, phosphates, hydrocarbons, and heavy metals, and enhance biogeochemical cycling—ultimately improving ecosystem resilience and water safety (Romantschuk et al. 2023).

In groundwater systems, bioremediation techniques often involve stimulating native microbes with nutrients or introducing specialized strains to accelerate contaminant breakdown (Wu and Yin 2020).

##### Built environment microbiome management

Designing buildings and ventilation systems to promote beneficial microbial exposure involves creating indoor environments that support microbial diversity and reduce the dominance of harmful pathogens. This approach recognizes that exposure to a wide range of non-pathogenic microbes, especially early in life, can help regulate immune responses and lower the risk of allergies, asthma, and infectious disease transmission. By incorporating natural materials, optimizing airflow, and allowing controlled interaction with outdoor microbiomes, architects and engineers can shape healthier built environments that align with public health goals and the One Health framework (Joseph et al. 2025).

For example, designing buildings and ventilation systems to promote beneficial microbial exposure, reducing the risk of allergies, asthma, and infectious disease transmission (Gilbert and Stephens 2018).

##### Microbiome surveillance in zoonotic disease prevention

Microbiome surveillance in zoonotic disease prevention involves monitoring microbial communities in animals, humans, and the environment to detect early signs of pathogen emergence and transmission. This approach is central to the One Health framework, which recognizes the interconnectedness of human, animal, and environmental health. By analyzing shifts in microbiome composition, especially in wildlife and livestock, we can identify potential reservoirs of zoonotic pathogens and assess risks before outbreaks occur (Carrera-Faja et al. 2025).

Microbiome-based surveillance offers several advantages over traditional methods. It enables the detection of both known and novel pathogens, provides insights into host–pathogen interactions, and supports predictive modeling for disease emergence and pandemic preparedness (Haraoui and Blaser 2023).

#### Future of microbiome restoration

The microbiome restoration strategies outlined above reveal the growing potential to rebalance disrupted biological systems. However, their effective implementation requires nuanced understanding of microbial ecology, host–microbe dynamics, and the surrounding environmental context.

The future of microbiome restoration is moving beyond traditional approaches toward precision, personalization, and integration with other technologies in the ecosystem (Coban et al. 2022, Gao et al. 2023, Abdulsalam et al. 2024). Emerging strategies are increasingly informed by systems biology, synthetic ecology, and AI-driven tools (Hernández Medina et al. 2022).

### Ecotron: experimental station for interdisciplinary studies

In the context of the One Health approach, ecotrons, which enable researchers to replicate natural ecosystems under controlled conditions, offer a fundamental tool for understanding how microbiomes contribute to ecosystem resilience and consequently to human well-being. In ecotrons, a variety of environmental variables, such as temperature, humidity, and microbial communities, can be changed, while ecosystem processes, such as gas exchange, nutrient cycling, and microbial activity, can be continuously monitored and manipulated (Granjou and Walker 2016). In this way, ecotrons provide powerful systems for investigating soil and plant microbiomes across diverse scenarios, including those related to global environmental change (Roy et al. 2021) and incorporating more complex multitrophic setups with microbial predators (Schmidt et al. 2021). In addition, novel technologies, including metabarcoding and environmental simulations integrated with ecotron platforms, can disentangle complex soil-plant-microbe interactions (Singer et al. 2021). This capability supports the development of microbiome-based interventions, such as enhancing beneficial microbial consortia to improve plant productivity, strengthening disease resistance, and aid in pollution mitigation. By fostering collaboration among microbiologists, ecologists, and agronomists, ecotrons generate actionable insights for managing soil and plant microbiomes. These insights contribute to mitigating (zoonotic) disease risks, improving food safety and security strategies, and adapting to global environmental changes offering transformative potential for the One Health approach (Granjou and Walker 2016).

## Enhancing ecosystem health through complementary strategies

### Groundwater ecosystem health: integrating a critical but overlooked element in the broader health framework

Groundwater is the world’s largest available store of freshwater, providing more than two billion people with drinking water and supplying approximately 40% of the global irrigation (de Graaf et al. 2019). Groundwater supports marine, freshwater, and terrestrial ecosystems by sustaining wetlands, rivers, and lakes, especially during dry periods.

Many plant and animal species rely on these groundwater-fed ecosystems for their survival. By maintaining the water levels in these habitats, groundwater helps preserve biodiversity and prevent the loss of species that depend on consistent water availability. Additionally, groundwater influences soil moisture, and in this way, it supports diverse ecosystems. Further, groundwater acts as a natural buffer against climate change by mitigating the effects of extreme weather events, such as droughts and floods. For example, during droughts, groundwater serves as a vital water source for both ecosystems and human communities, maintaining water supply when surface water sources are depleted. Conversely, during periods of heavy rainfall, groundwater systems can absorb excess water, reducing the risk of flooding and soil erosion (Esteban et al. 2021, Saccò et al. 2024).

Groundwater is also vital for agriculture, providing irrigation water that supports food production. Sustainable management of groundwater resources ensures that agricultural lands remain productive, helping to meet the food demands of a growing global population. This, in turn, contributes to food security and economic stability services (Griebler and Avramov 2015).

The pressure on groundwater can be mitigated by using alternative water sources such as harvested rainwater from roofs, harvested stormwater, reclaimed wastewater, graywater, and captured condensate (Manyuchi et al. 2018).

To enable all the essential functions of groundwater, we must ensure its quality. For this purpose, a Groundwater Quality Index (WQI) is utilized. This index incorporates various physical, chemical, and biological parameters, including the presence of coliform bacteria and other pathogens. The WQI provides a comprehensive assessment of groundwater suitability for different uses by converting complex water quality data into a single, understandable index. Zhong and colleagues (2023) emphasize the importance of assessing the groundwater microbiome as a more comprehensive approach to evaluate anthropogenic impact and ecosystem health (Zhong et al. 2023). Indeed, there is an urgent need to improve the biological monitoring of groundwater to measure the native microbiome, not just coliforms and pathogens (Fillinger et al. 2023, Di Lorenzo et al. 2024).

### Nanostructurome: hidden communication and responses at the nanoscale

The study of cell-to-cell interactions mediated by nanostructures holds immense importance for the One Health approach, as it delves into the intricate and largely unexplored landscape of the communication at the nanoscale. By examining how sub-micron cellular structures convey matter and information between cells, we gain deeper insights into fundamental biological processes on how cells interact within and across different biological systems, including humans, animals, and the environment. The expression “nanostructurome” stands for knowledge on these nanostructures, which is yet largely obscure but will with time become filled with information.

Extracellular particles (EPs) are nanostructurome subjects that can transport a variety of cargo to adjacent or remote cells (Lenzini et al. 2020, Welsh et al. 2024). Biologically active EPs are produced by all types of cells, including microorganisms, containing those with thick cell walls (Fang et al. 2022) (figure 6). Depending on the type, EPs can be resistant to attack by degradative enzymes thus enabling long-distance delivery, specificity in host-cell targeting, and evasion of the host immune system (Bonnington and Kuehn 2014). They were found to mediate and trigger inter- and intra-cellular communication, biofilm formation, antibiotic resistance, stress responses, toxin delivery, and the transfer of virulence factors (Woith et al. 2019). A growing body of evidence suggests that exosome-induced changes in EP profile could regulate, for example, the aging process, both at the cellular and organismal levels (Monti et al. 2021).

**Figure 6.**
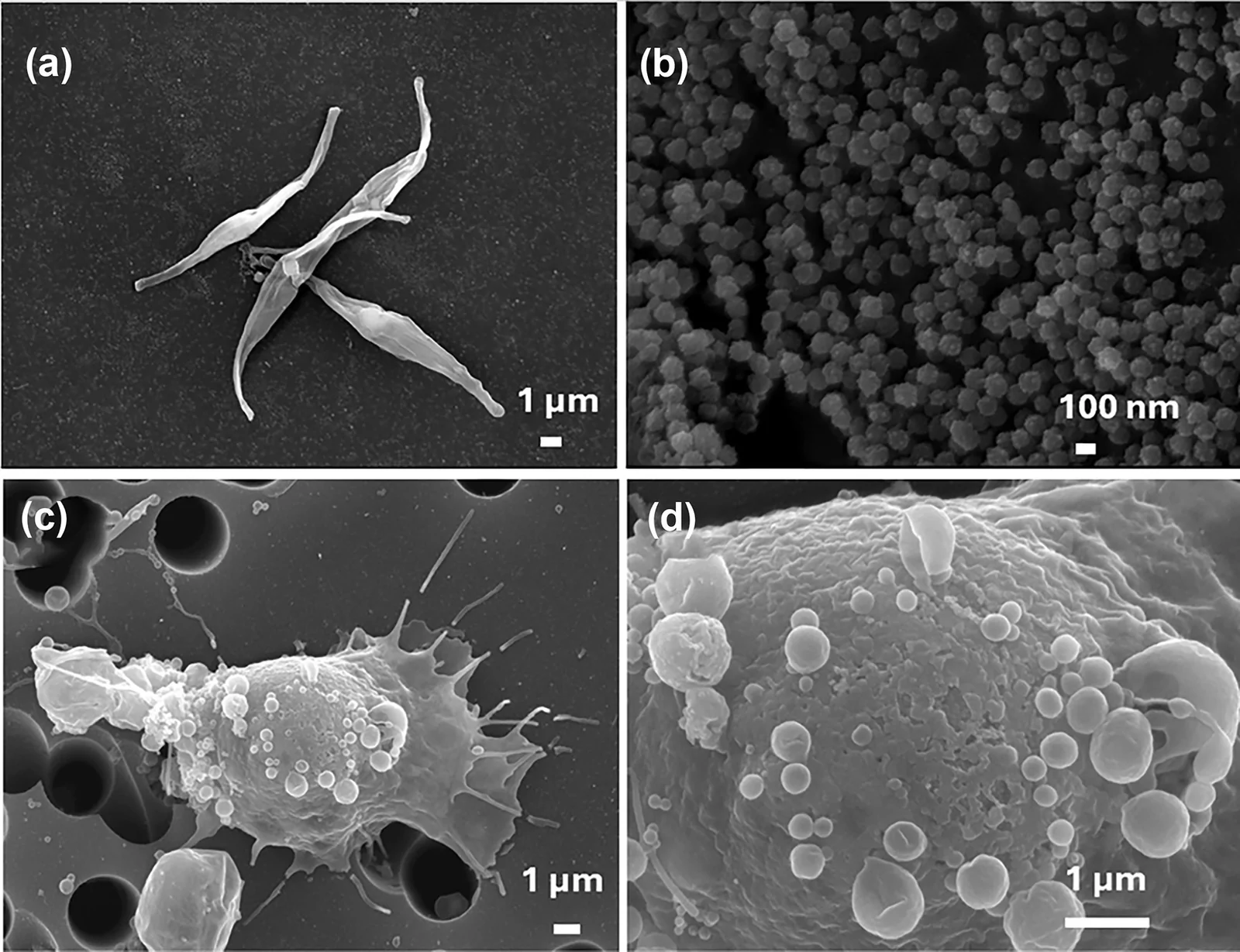
Microalgae *Phaeodactylum tricornutum* with numerous EPs in the background (upper left) and EPs at higher magnification (upper right) (Bedina Zavec et al. 2024). Stressed *Mediterranean mussel (Mytilus galloprovincialis)* blood cells releasing EPs (lower left at lower magnification and lower right at higher magnification) (by Matej Hočever, IMT, Ljubljana).

The exploration of the nanostructurome is a significant step toward advancing the One Health approach by fostering a comprehensive understanding of health and disease mechanisms at the cellular. For example, the nanostructurome study can contribute to monitoring environmental health by identifying how pollutants and other environmental factors affect cellular communication and health (Monti et al. 2021, Škufca et al. 2022).

Recent studies have identified plant-derived EPs to play a crucial role in modulating plant–microbiome interactions. By shaping the rhizosphere microbiome, enhancing beneficial microbes while suppressing pathogens, EP-based approaches contribute to healthier soil ecosystems, improved nutrient cycling, and greater resistance to biotic stress. This microbiome-informed strategy aligns with the principles of regenerative agriculture and offers a biologically precise alternative to conventional inputs (Liu et al. 2021).

For example, green microalgae such as *Chlorella vulgaris* and *Scenedesmus obliquus* produce EPs enriched with peptides and metabolites that inhibit the growth of *Pseudomonas syringae* and *Fusarium oxysporum*, two major crop pathogens (Picciotto et al. 2022). These vesicles can be applied as foliar sprays or soil amendments to reduce disease incidence without disrupting beneficial microbial communities. Additionally, algal EPs can be used to deliver RNA interference molecules to pests or pathogens, offering a non-GMO alternative to genetic modification (Picciotto et al. 2021).

In the context of antimicrobial resistance, algal EPs offer a dual advantage: they can outcompete resistant strains through quorum sensing disruption and enhance the efficacy of existing biocontrol agents. Their biodegradable nature and compatibility with organic farming systems make them ideal candidates for next-generation crop protection strategies, and support environmentally friendly farming systems.

### Engaging with citizen science

Citizen science is an approach that could significantly expand One Health Community and engage stakeholders. The Manhattan Principles, formulated in 2004 and updated as the Berlin Principles in 2019, aim to enhance cross-sectoral and transdisciplinary health surveillance. They seek to improve coordination among governments, NGOs, healthcare organizations, academia, industries, and other stakeholders. Additionally, these principles integrate ecosystem health into the One Health approach, addressing pressing issues such as climate change and antimicrobial resistance.

The advantage of engaging and collaborating with a diverse range of stakeholders, including health diplomacy and advocacy groups, the private sector, academia, national governments, civil society, and individuals, is to identify public needs, address local priorities, or empower community-based organizations. This highlights the importance of using participatory models to involve nonprofessionals in scientific research (Eleta et al. 2019). For instance, citizen science projects can monitor environmental factors such as air and water quality, which are crucial for preventing zoonotic diseases and ensuring ecosystem health (Remmers et al. 2024).

The One Health interventions that use citizen science to improve human, animal, and ecosystem health could identify new domains of innovative practice that supports resilience in the face of climate change and environmental health hazards. An example of good practice in participatory microbiome research is the Microsetta Initiative, which aims to collect and analyze human microbiome data from participants around the world. The initiative builds on previous projects like the American Gut and British Gut Projects, expanding its reach to create population-specific microbiome references (The Microsetta Initiative 2025).

### Advancing One Health with social science perspective

Since the early 1980s, the WHO (Alma-Ata Declaration 1978, Ottawa Charter on Health Promotion 1986) emphasized the multiple determinants of population health, leading to the concept of “Health in All Policies” (HiAP), which integrates health considerations into all areas of public policy. It recognizes that health outcomes are highly influenced by social determinants, including economic, social, and environmental factors, which are not the sole responsibility of public health departments. Therefore, HiAP emphasizes intersectoral collaboration in developing interventions that improve the overall health of the population and reduce health inequalities. Environmental factors are inextricably linked to the social determinants of health, which the WHO defines as “the conditions in which people are born, grow up, work, live and age, as well as the broader set of forces and systems that shape the conditions of daily life” .

Michel Serres and Luc Ferry, both prominent philosophers, have further elaborated on the role of social determinants in the health discourse. Luc Ferry emphasized the need for pragmatic, human-centered action, whereas Michel Serres recognized the broader, ethical interdependence of all life. Both perspectives form the basis of the Social-Ecological System Health (SESH) framework, which aims to reconcile human needs with an ethical commitment to ecological integrity. SESH integrates the interdependence of different system elements, including people, institutions, economies, cultures, and the environment, all of which form an integral part of the ecosystem (De Garine-Wichatitsky et al. 2021).

The SESH framework emphasizes three main dimensions: (a) the health of humans, animals, and plants; (b) the health of the environment, encompassing both biophysical environment health and human environment health; and (c) the overarching health of the social-ecological system (SES) itself. Together, these components address public and veterinary health, biodiversity conservation, ecosystem integrity, governance, social capital, and other social determinants of health, thus linking resilience and sustainability. By fostering interdisciplinary collaboration, the SESH concept provides an approach to address interlinked health and environmental problems in biomedical, ecological, socio-cultural, and economic domains. Taking all these aspects into account, Quenan and colleagues (2017) provided a roadmap to a One Health agenda 2030. They propose two key One Health objectives: recognizing humans as part of a complex ecosystem (intrinsically linked to its health, requiring respect for natural origins, reconnection with nature, and adherence to ecological boundaries) and acknowledging dependence on ecosystem services (addressing the negative impacts of past development). To achieve these objectives, Quenan and colleagues (2017) proposed a transdisciplinary collaboration, involving experts from various fields working together.

One Health scholars have emphasized the need for an inter- or transdisciplinary approach (Lapinski et al. 2022). None of the scientific disciplines or stakeholders should be underrepresented in discourse formation (Drobne et al. 2023). To develop effective interventions, it is important to incorporate novel communication and education strategies that use technologies such as mobile apps and social media to promote behavior change and inform decision-making processes that benefit human, animal, and ecosystem health (Mair et al. 2023).

### Microbiome literacy

Public understanding of microbiome science is hindered by misinformation and limited accessible educational resources (Schweitzer et al. 2023). Thus, in the context of One Health, microbiome literacy is essential to enhance public and academic understanding of microbiome science and foster informed decisions for sustainable health and environmental stewardship. Joint multidisciplinary interventions that integrate microbiome stewardship into One Health education and policy are essential for a sustainable economy and resilient systems. Integrating microbiome-related content into school, university, and vocational curricula can encourage interdisciplinary learning and prepare future professionals to connect scientific data with policy and practice. Cultivating and promoting microbiome literacy through public health education can empower communities to make informed decisions about hygiene, diet, and medicine, reinforcing the need for integrated, cross-sectoral approaches. For instance, the “Microbiome & Health” online course developed by Schweitzer and colleagues (2023) introduces core microbiome concepts and their relevance to human, plant, and environmental health. The curriculum aligns with the One Health framework and the UN Sustainable Development Goals, demonstrating how education can bridge scientific understanding and community action.

### A need for new professional profiles and novel education strategies

Successful implementation of the One Health concept requires a shift in thinking from “disease care” to prevention. This calls for preparing professionals to tackle complex health challenges together and to promote a holistic approach to health that recognizes people, animals, plants, and ecosystems as interconnected while also considering the social determinants of health and well-being (Hristovski et al. 2010). Education, from early childhood through adulthood, plays a particularly important role in this transformation (Barrett et al. 2011). Problem-based learning, team-based learning, simulation-based education, case-based learning, and interdisciplinary workshops and seminars should be implemented into educational practice (Cai et al. 2024).

In adult education, microdiploma programs can incorporate One Health topics using blended and microlearning approaches (Kohnke et al. 2024) as a new learning concept and powerful tool for lifelong learning (De Rosa et al. 2024). Embedding these principles into standard curricula across disciplines from science, technology, engineering, and mathematics to the humanities will help effectively address future health challenges (Rabinowitz et al. 2017, Cai et al. 2024). To ensure that future professionals can implement the One Health approach, education must extend beyond disciplinary boundaries and promote systems thinking that connects environmental, social, and biomedical domains. Curricula should build competencies to interpret complex information and apply it to policy, management, and innovation. Strengthening collaboration among the natural and social sciences through lifelong learning will further support evidence-based and integrative One Health strategies.

## Conclusions

Currently, health is at the forefront of both political and public discourse. In 2022, the new definition of One Health shifted the traditional focus from zoonoses and antimicrobial resistance to the full spectrum of health, including ecosystem health. It recognizes that the health of humans, domestic and wild animals, plants, and the ecosystems is closely linked and interdependent. In addition, the integration of health and SES created the framework for the SESH. Despite broad international recognition and support, the operationalization of One Health remains a challenge.

In this paper, we propose leveraging microbiome analyses to strengthen the One Health approach. Quantitative metrics form the basis for indicators that characterize microbiome status such as stress, resilience, stability, plasticity, robustness, homeostasis, functional redundancy, and dysbiosis, which serve as proxies for microbial health across domains, including the human and animal gut, food systems, soil, water, and the built (urban) environment (figure 7). These insights support targeted interventions ranging from microbiome-based therapies to environmental restoration and integrated surveillance systems, fostering health across humans, animals, plants, and ecosystems.

**Figure 7.**
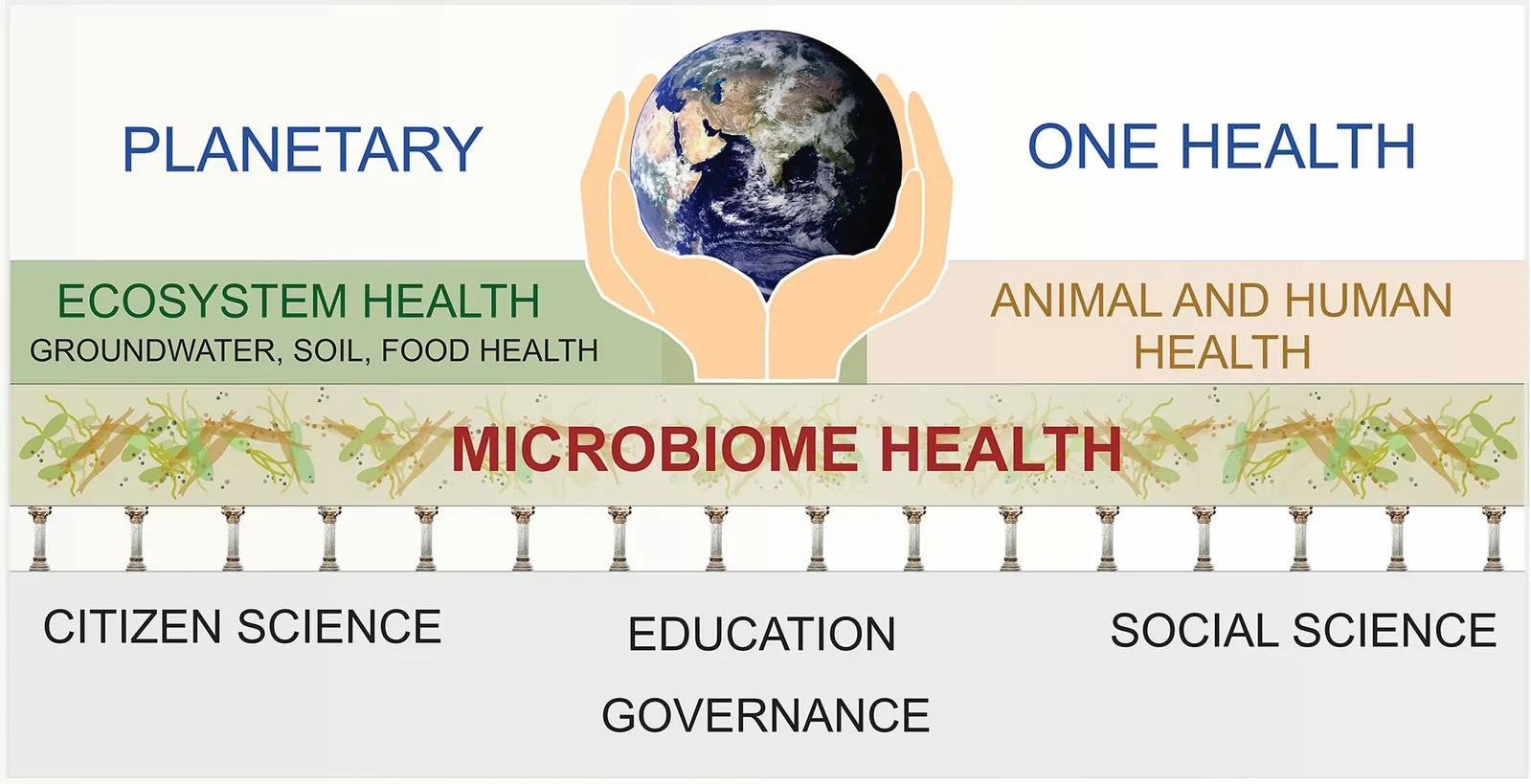
Interdependence between human, animal, and environmental health (BioRender-generated image).

However, long-term success in advancing One Health is dependent on microbiome-health literacy, innovative educational strategies, citizen science engagement, and support rooted in social science perspectives. Given that microbiological monitoring programs and data already exist, a critical first step toward implementing the One Health approach is to integrate these databases and start with coordinated, cross-sectoral action.
